# Mr. Henry Jeffreys on the Use of Cubebs in the Cure of Gonorrhœa

**Published:** 1821-09-01

**Authors:** 


*Practical Observations on the Use of the Cubebs, or Java Pepper, in the Cure of Gonorrhœa. With Cases.* By Henry Jeffreys, Esq. Senior Surgeon of the St. George's and St. James's General Dispensary; Assistant Surgeon to the Lock Hospital; and formerly a Surgeon in the Third Regiment of Guards. One vol. 8vo, pp. 58. Callow, London, 1821.

We doubt much the correctness of Mr. Jeffreys' position, that Gonorrhœa "has descended to us from a very remote antiquity." We are rather disposed to think that, like many other diseases, it has sprung up from a more recent cultivation of a polluted soil. However that may be, its treatment is often difficult and unsatisfactory both for patient and surgeon. It is a treacherous disease, often appearing cured, and often suddenly returning upon the application of very trifling causes. Its consequences, too, are not seldom of a distressing nature, particularly as regards the calibre of the urethra, and the facility of discharging those excretions and secretions that are destined to travel through that channel.

Cubebs and Capivi have, of late years, been noisy candidates for *specific* honours in the methodus medendi of Gonorrhœa, or what we think is a more proper term, Blennorrhagia. The contest is not so easily decided as the raising a professor to a chair; nor is is perhaps of so much consequence to the republic of medicine. No detailed account of Cubebs having been given to the world, Mr. Jeffreys has here given the result of that experience, which a public institution and private practice afforded him—his design being nothing more than to record this result for the benefit of others. This design is laudable, and we proceed to sketch out the execution of it.

Our author justly remarks, in some introductory observations, that patients, looking upon blennorrhagia as a complaint more interfering with their comforts or pleasures, than of much consequence in itself, can seldom be induced to acquiesce in the necessary confinement, or submit with resolution to the strict treatment and abstemious regimen necessary for its eradication. For this disobedience they generally sup sorrow in the end—but the worst of it is, that they have a very ready knack of shifting the blame from their own shoulders to those of their medical attendant.

Mr. Jeffreys regards gonorrhœa "simply as a local affection," and to be rarely, if ever, followed by constitutional, or what are called *secondary* symptoms, except where mercury has been resorted to. We have seen some constitutional symptoms, as eruptions, sore throats, and fever, follow gonorrhœa, where no mercury was used. Mr. Carmichael and others have seen the same thing. In respect to the treatment, by Cubebs, the attention of the profession was excited a few years ago by Mr. Crawford's paper in the Edinburgh Journal, and our author observes that, whatever may have been the success of others, in his own practice he found Cubebs "not only a very safe remedy, but, in the generality of cases, infinitely more useful and expeditious than any which has ever yet been introduced into practice for the cure of gonorrhæa." 10.

The Cubebs pepper is indigenous in Java, being found in the woods near Batavia. It is also met with in the Isle of France and other parts of the East. The berries grow in clusters, like currants, and when dried have a wrinkled surface, light brown colour, warm, pungent, aromatic, and slightly bitter taste, but much milder than common pepper. A tincture may be made (cubebs ℥iij. sp. vin. ten. Oj.) possessing all the virtues of the berry. Taken into the stomach, the Cubebs does not appear to produce any very particular or predominant action on the system. Like other spices it is warm and stimulant—in some constitutions it proves mildly aperient, "probably by giving tone and vigour to the bowels, in the same way as the celebrated Ward's paste." In others it has a contrary effect, and requires a laxative occasionally during its exhibition. In some persons to whom our author has administered it, head-ache and nausea were induced.

"When given in large doses, as a drachm and a half or two drachs of the powder four times in the day, it appears to increase the secretion of urine, which assumes a deeper tinge of colour than usual, is voided somewhat more frequently and in larger quantity at a time, and acquires a peculiar and slightly aromatic odour, which is not unpleasant." 16.

Of the modus agendi of this medicine in gonorrhœa, Mr. J. professes his ignorance. We might all say the same thing respecting the greater number of medicines in the pharmacopœia. Its agency appears to Mr. Jeffreys to resemble that of copaiba.

"It possesses, however, what may justly be called a specific power in most constitutions, especially when administered in the early and acute form of the disease. It moderates the inflammatory and most painful symptoms, and suppresses the quantity of the discharge in a shorter time, and with more certainty, than any other remedy with which I am acquainted. I have reason to believe, that its exhibition is rarely, perhaps never, followed by any of those consequences, which so often take place under the more ordinary modes of treatment, when the discharge is suddenly checked, such as irritation in the bladder, strangury, swelled testicle, &c. It has not, at least, occurred to me to witness any of these accidents in my experience of the remedy; and, as far as I have been enabled to observe, it is in the more inflammatory forms of the disease in which its efficacy is most certainly displayed." 18.

The good effects of Cubebs, in cases where it is serviceable, commonly begin to manifest themselves within 48 hours after the first dose. Unless some material relief is obtained in the course of five or six days, "its continued administration will rarely be attended with advantage." The period of cure, in successful cases, has varied from two or three days to a fortnight or more. In many cases, Mr. J. observes, it has proved to be of considerable efficacy where other remedies had been tried in vain, and where the disease was of some standing.

"Out of the first twenty-one cases in which I prescribed it, taken indiscriminately as they occurred, fourteen were cured, four relieved, and in three it failed. Of those in which it appeared to afford only a partial relief, the want of complete success may fairly be attributed to some irregularity or inattention in the patients' mode of living, or to a carelessness in taking the medicine. Nevertheless, one important advantage has appeared to me to be derived in these cases from the administration of the Cubebs, which is, that the symptoms afterwards yielded with greater facility to copaiba than under ordinary circumstances." 19.

Although there appears no good reason why Cubebs should not be combined with other medicines in gonorrhœa, yet

Mr. Jeffreys did not find that any material advantage was derived from the combination. When the more urgent symptoms were allayed, the cure appeared to be accelerated by a mildly astringent injection. Gleet, one of the most common and intractable sequela of gonorrhœa, under ordinary circumstances, will be found, Mr. J. thinks, a less frequent consequence of the disease when it has yielded to Cubebs. Mr. J. administered the medicine in water, the patient being, in every instance, enjoined rest and an abstemious diet—an injunction but seldom strictly attended to. The dose, we observe by the cases, was, from a drachm to a drachm and a half, three or four times a day.

"Upon the whole, I trust I have adduced sufficient facts to convince the candid reader, that, although the Cubebs pepper may not be entitled to the rank of a *specific* in the cure of gonorrhœa, it has nevertheless a better claim to that distinction than any medicine that has hitherto been made use of in the treatment of the complaint; and that, at any rate, it furnishes the surgeon with a remedy of great power, which may be applied in all cases without risk, and in many with a fair chance of a *speedy* and *permanent* cure." 22.

The cases, which are 27 in number, appear to be faithfully detailed, the unsuccessful as well as the successful—a mode of conduct which is very praiseworthy. Our author, at the end of the cases, informs us that, as far as he has hitherto been able to ascertain, "there is no symptom attending gonorrhœa which contra-indicates the use of Cubebs." He has not scrupled to prescribe it in all the stages, and under all the circumstances of the complaint, without seeing any reason to repent of so doing. He considers it absolutely necesssary that the Cubebs should be freshly powdered, and free from adulteration. It is of great importance also, that the medicine should not be left off too soon after the symptoms have disappeared.

As it is not consistent with our plan, or the space allotted to this article, to enter on any analysis of the cases, we refer our readers for further information, in detail, to the work itself, which is characterized by plainness of narrative, freedom from affectation, and apparent candour of detail. These are all which we can look for, in a publication of this description, and all, we believe, which the author himself aims at.

